# Supplementary material for: Early Administration of Tolvaptan Can Improve Survival in Patients with Cirrhotic Ascites
Source: J Clin Med. 2021 Jan 14;10(2):294. doi: 10.3390/jcm10020294 (PMC7830941; doi:10.3390/jcm10020294)
Supplement: Supplementary file 1 [file jcm-10-00294-s001.pdf]

Supplementary Material

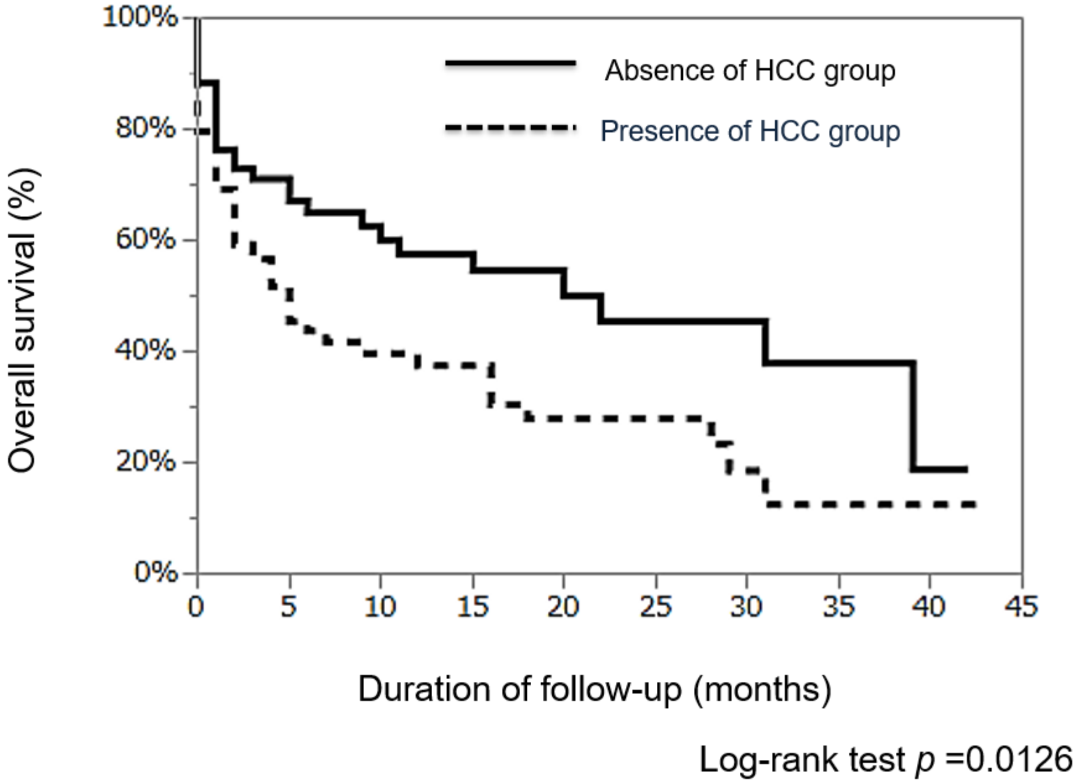

Supplementary Figure S1. Comparison of cumulative survival rates between the tolvaptan group with HCC and without HCC.
